# Supplementary material for: Tyrosine Kinase Inhibitor Profiling Using Multiple Forskolin-Responsive Reporter Cells
Source: Int J Mol Sci. 2023 Sep 8;24(18):13863. doi: 10.3390/ijms241813863 (PMC10530646; doi:10.3390/ijms241813863)
Supplement: Supplementary file 1 [file ijms-24-13863-s001.zip › ijms-2532424-supplementary.pdf]

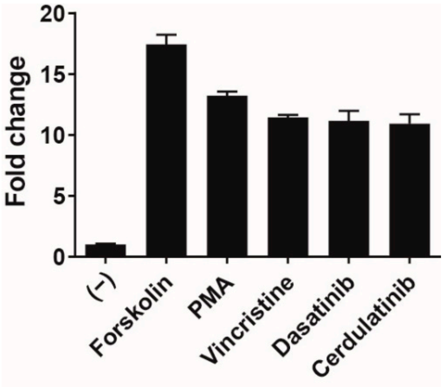

Figure S1. A forsklin-responsive cell clone (#F1) showing pleiotropic compound-responsiveness. Cells were treated with 0.5 μM of the indicated reagents overnight, and luciferase assay was performed. The graph shows the mean ± SD of three samples.

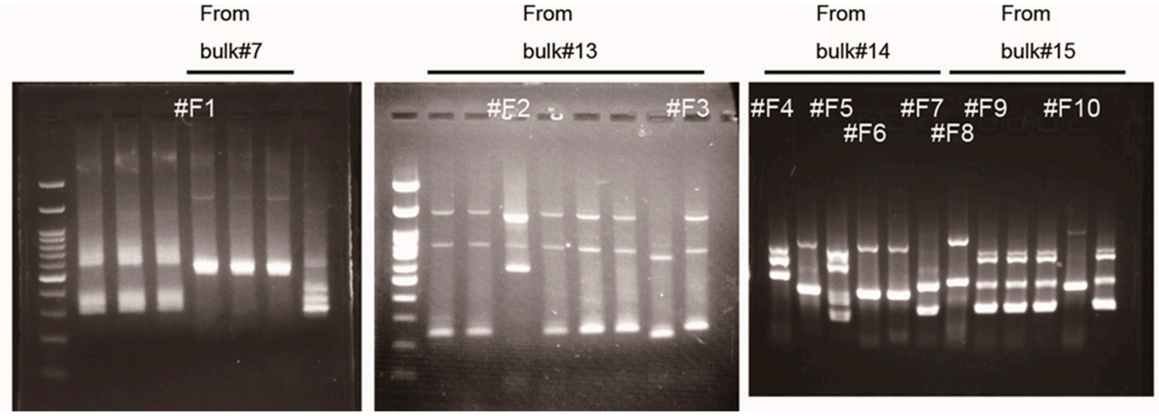

Figure S2. Analysis to eliminate duplicate clones derived from the same bulk cells. Splinkerette PCR analysis was performed as described in Ishikawa (2018). Differences in band patterns among these clones suggest that the transposon vector insertion sites are different. Indicated independent clones correspond to the clones shown in Figure 1b.

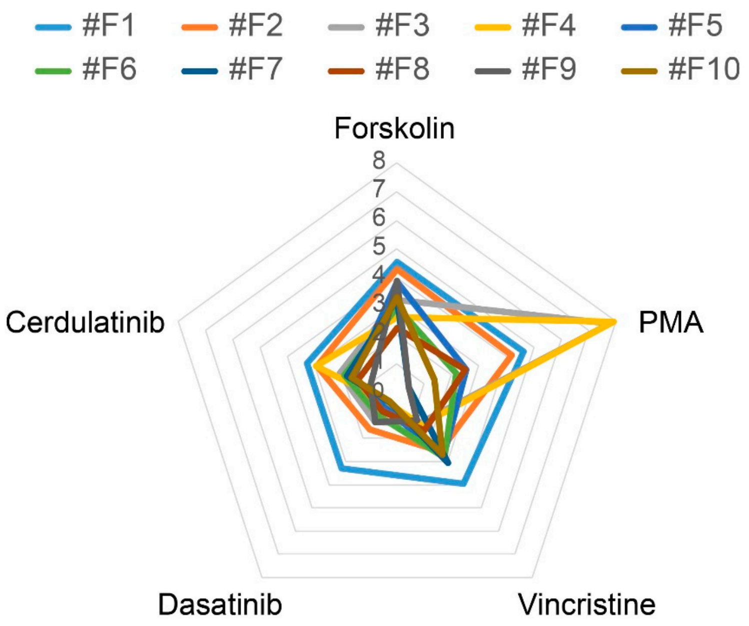

Figure S3. Radar chart display of the values from the luciferase assay in Figure 2. The data in Figure 2b was plotted on a radar chart with each reagent as the axis. Figure 3 is a separate display of this figure for each clone.

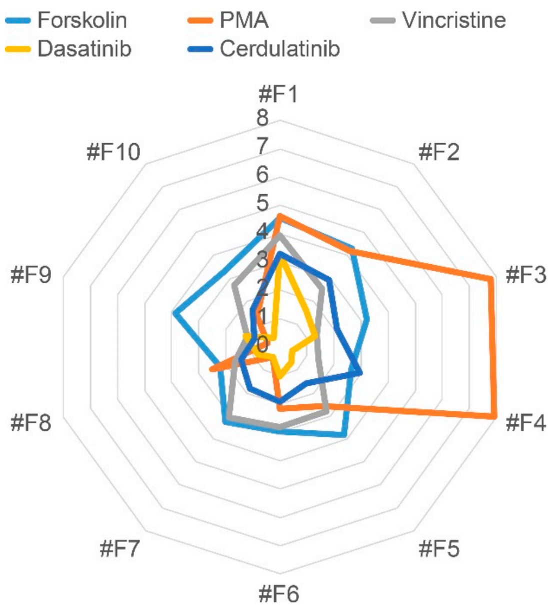

Figure S4. Radar chart display of the fold change value. The data in Figure S3 were replotted with each clone as the axis. Figure 4 is a separate display of this figure for each reagent.

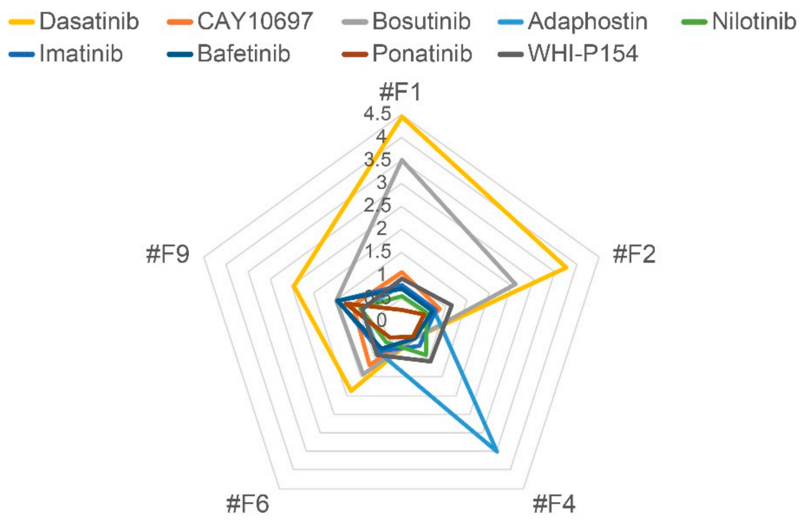

Figure S5. Unique profiling of TKIs using five forskolin-responsive clones. Data from Figure 6b were converted to a radar chart with each clone as the axis. Figure 6c is a separate display of this figure for each reagent.

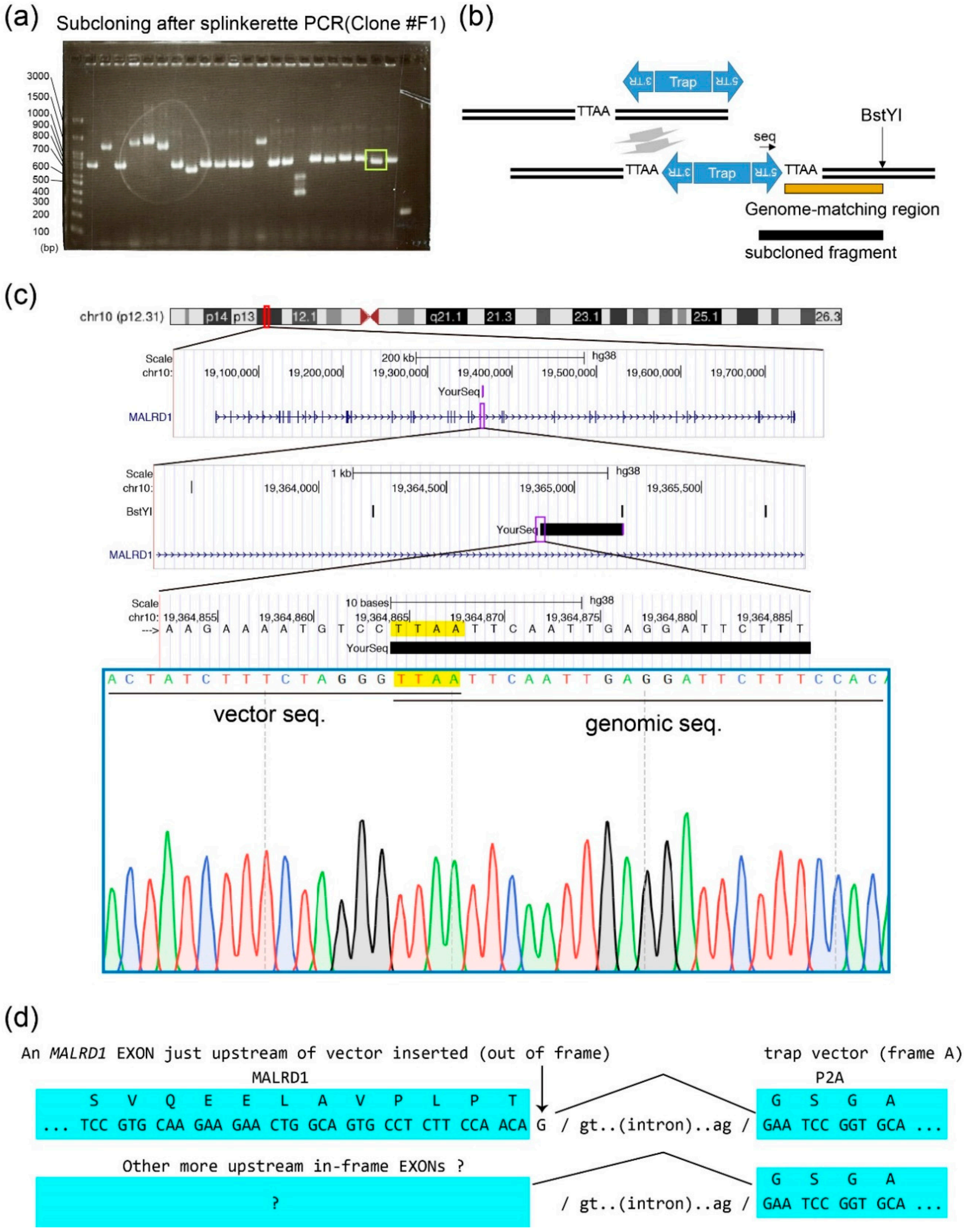

Figure S6. Responding gene identification for #F1 reporter clone by splinkerette PCR. (a) PCR band in Figure S2(#F1) were subcloned into pBluescript II SK(−) vector and colony PCR analysis was conducted followed by electrophoresis using 2% agarose gel. The clone marked by the yellow square is the one for which the gene was identified after sequencing. (b) Schematic diagram of a DNA region analyzed by splinkerette PCR for trap vector's genomic integration site. TTAA is a short recognition sequence of the *piggyBac* transposase (Yusa et al. 2011). (c) Sequence was analyzed with UCSC genome browser to identify where the transposon vector was inserted into

the genome. A sequence that are likely to be recognized by the *piggyBac* transposase is marked in yellow. Sequence and its wave data, and vector and genome sequence positions are shown in the panel below. (d) Predicted transcripts fused to the reporter inferred from the analysis. According to the general rule, if the exon of *MALRD1* directly above the inserted intron region is fused by splicing, the reading frame does not match that of the reporter gene, suggesting that a more upstream in-frame exon was probably fused by splicing. There are 10 upstream in-frame exons in *MALRD1*.

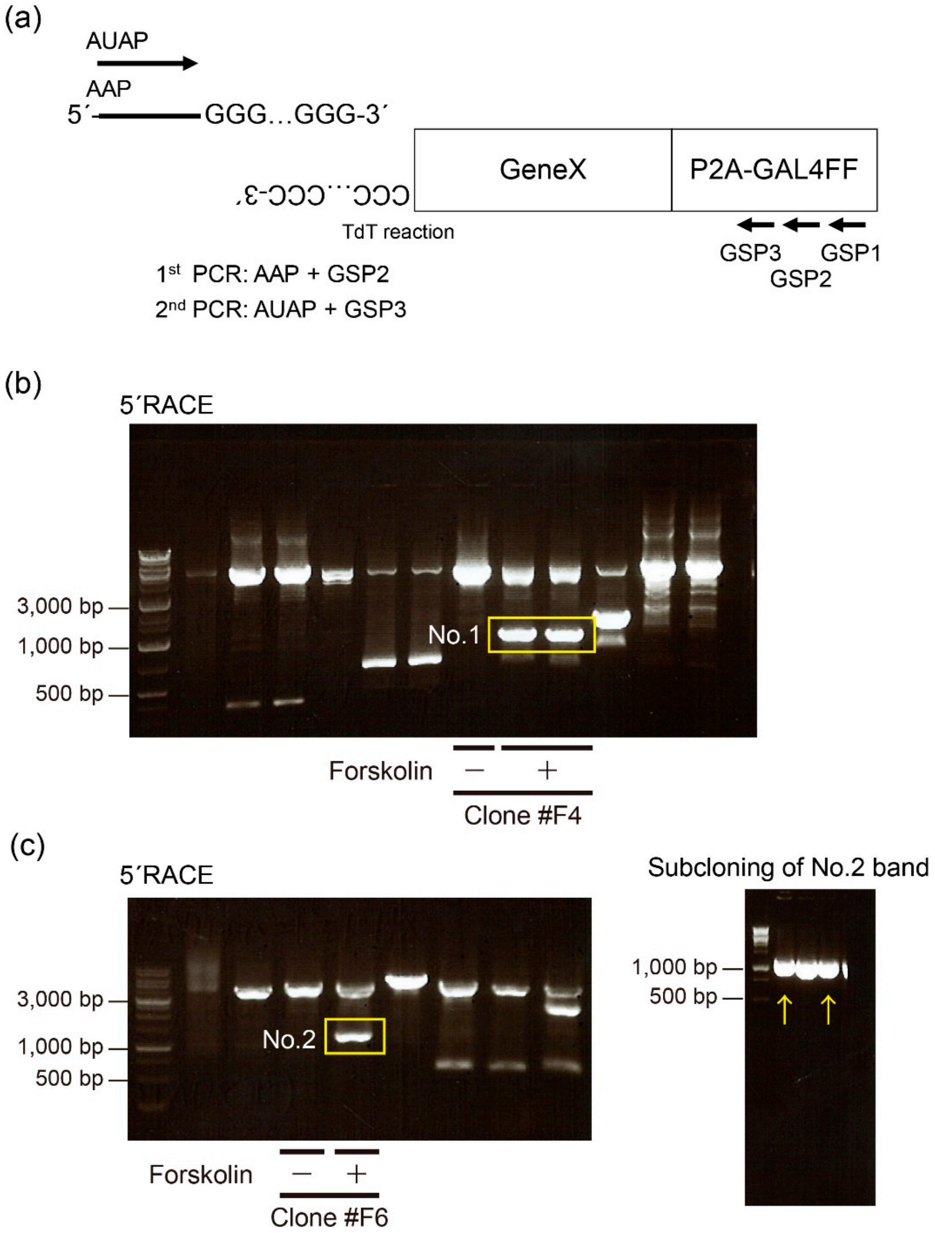

Figure S7. Responding gene identification for reporter clones by 5′RACE. (a) Schematic diagram of 5′RACE including primer positions, designed to identify gene X whose transcript was fused to *P2A-GAL4* by a splicing mechanism. TdT, terminal deoxynucleotidyl transferase. (b, c) After 1.5% agarose gel electrophoresis for nested PCR in 5′RACE, DNA bands (yellow rectangular) that specifically appeared in the sample treated with forskolin (+) were collected. Only clone samples in which genes were identified are presented. (b) The DNA band No.1 derived from

clone #F4 were purified and directly analyzed for sequence. (c) No.2 derived from clone #F6 (left picture) was subcloned into the pBluescript II SK(−) vector, and then colony PCR was conducted followed by electrophoresis using 2% agarose gel (right picture). The subcloned plasmids corresponding to the template of yellow arrows were used for analysis of its sequence (the two were turned out to be identical sequence).

(a) Clone #F4_5´RACE

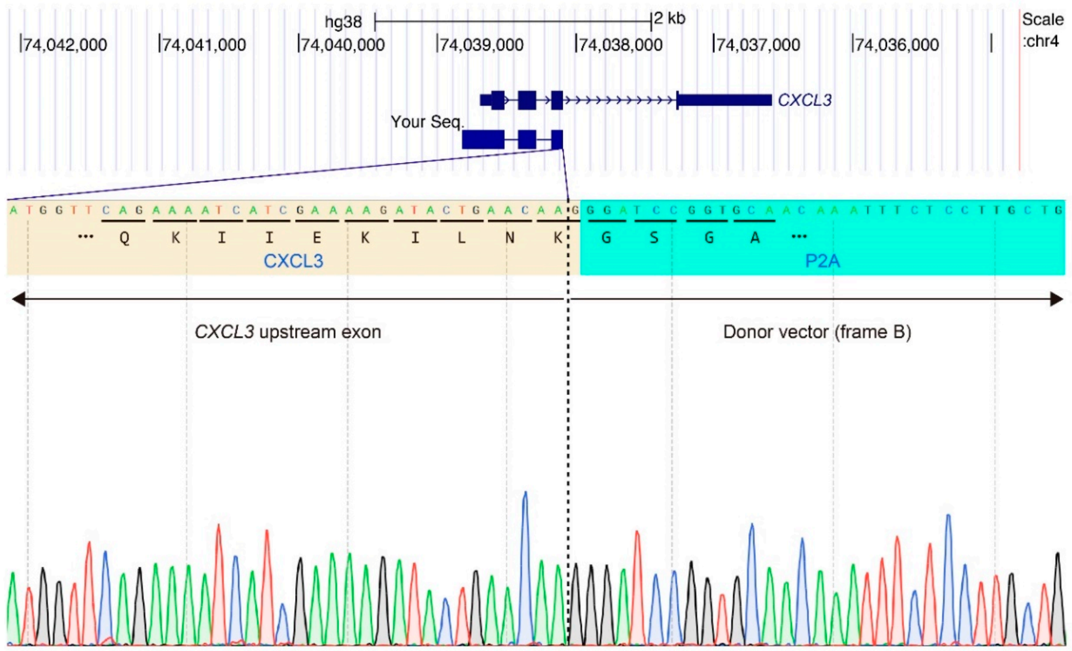

(b) Clone #F6_5´RACE

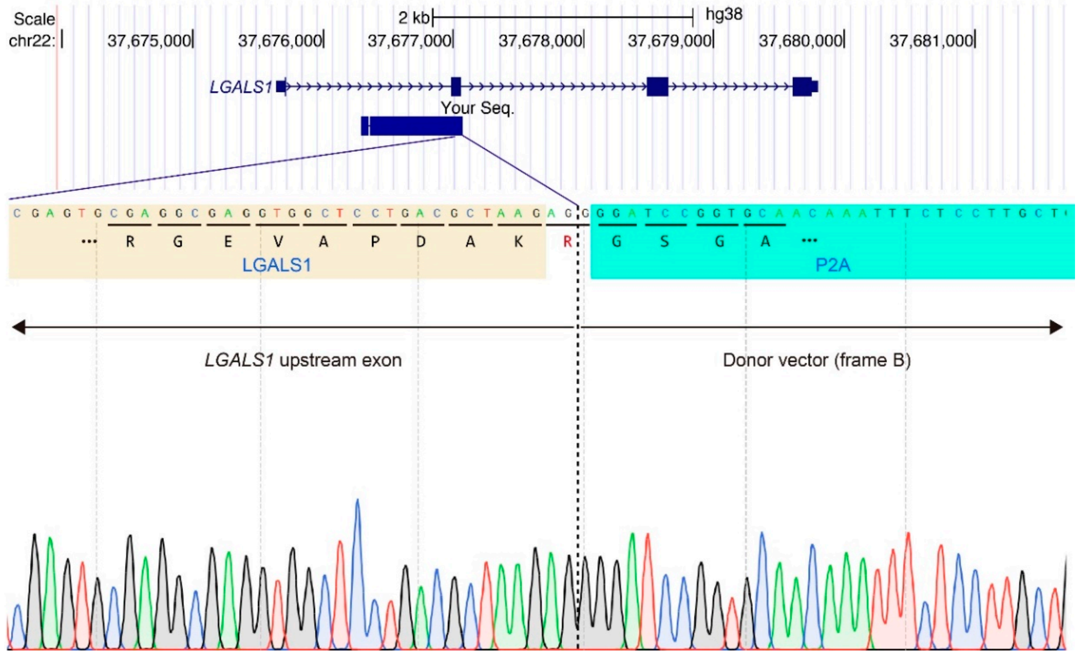

Figure S8. Mapping of sequence of the DNA fragment or subcloned plasmid obtained from 5′RACE of #F4(a) #F6(b) clones (Figure S7). Along with sequence and wave data, it showed how *P2A-GAL4FF* was fused with the endogenous gene. Note that the transcript of *LGALS1* fused to *P2A-GAL4FF* seems to have a different transcription start site, not found in the database. Since the translation start site (ATG) of *LGALS1* is not found on the *P2A-GAL4FF* reading frame, it is possible that GAL4FF is translated directly,

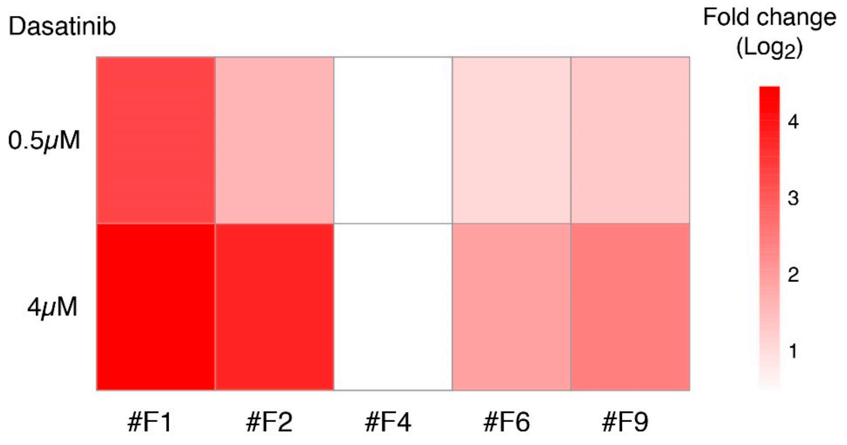

Figure S9. Comparison of the response of each reporter clone to different concentrations of dasatinib. Data for luciferase assay for dasatinib in Figure 2a and 6a are re-presented in heatmap. Here, there is a significant difference in responsiveness depending on the dose of dasatinib, especially for #F2. Adding such differences in dose dependency to the profiling may help us to understand the properties of the drug more deeply.
